# Direct comparisons of bisulfite pyrosequencing versus targeted bisulfite sequencing

**DOI:** 10.17912/micropub.biology.000444

**Published:** 2021-08-19

**Authors:** Dillon E. King, A. Clare Sparling, Rashmi Joglekar, Joel N. Meyer, Susan K. Murphy

**Affiliations:** 1 Nicholas School of Environment, Duke University, Durham NC; 2 Department of Obstetrics and Gynecology, Duke University, Durham NC

## Abstract

DNA methylation is an important epigenetic mechanism involved in proper genome function. Bisulfite pyrosequencing (PSQ) is a commonly used technique to quantify DNA methylation. Although very accurate, bisulfite pyrosequencing can be expensive and time consuming for large-scale quantitative DNA methylation analysis at the single nucleotide level. High throughput DNA methylation sequencing has the potential to address these limitations, but its comparability to other methylation detection methods has not been well studied. We compared QIAseq Targeted Methyl Panel technologies (QMS) and PSQ by analyzing four CpG sites within four genes involved in neurodevelopment*. *QMS and PSQ had an average 5.6% difference in the detected level of DNA methylation for the same four CpG sites. However, we observed a strong correlation in the levels of methylation across all four CpG sites between the two technologies. These findings demonstrate the comparability of QMS relative to PSQ in the ability to accurately quantify DNA methylation at specific CpG sites.

**Figure 1. DNA methylation measured at the same CpG sites and in the same samples using two technologies:**
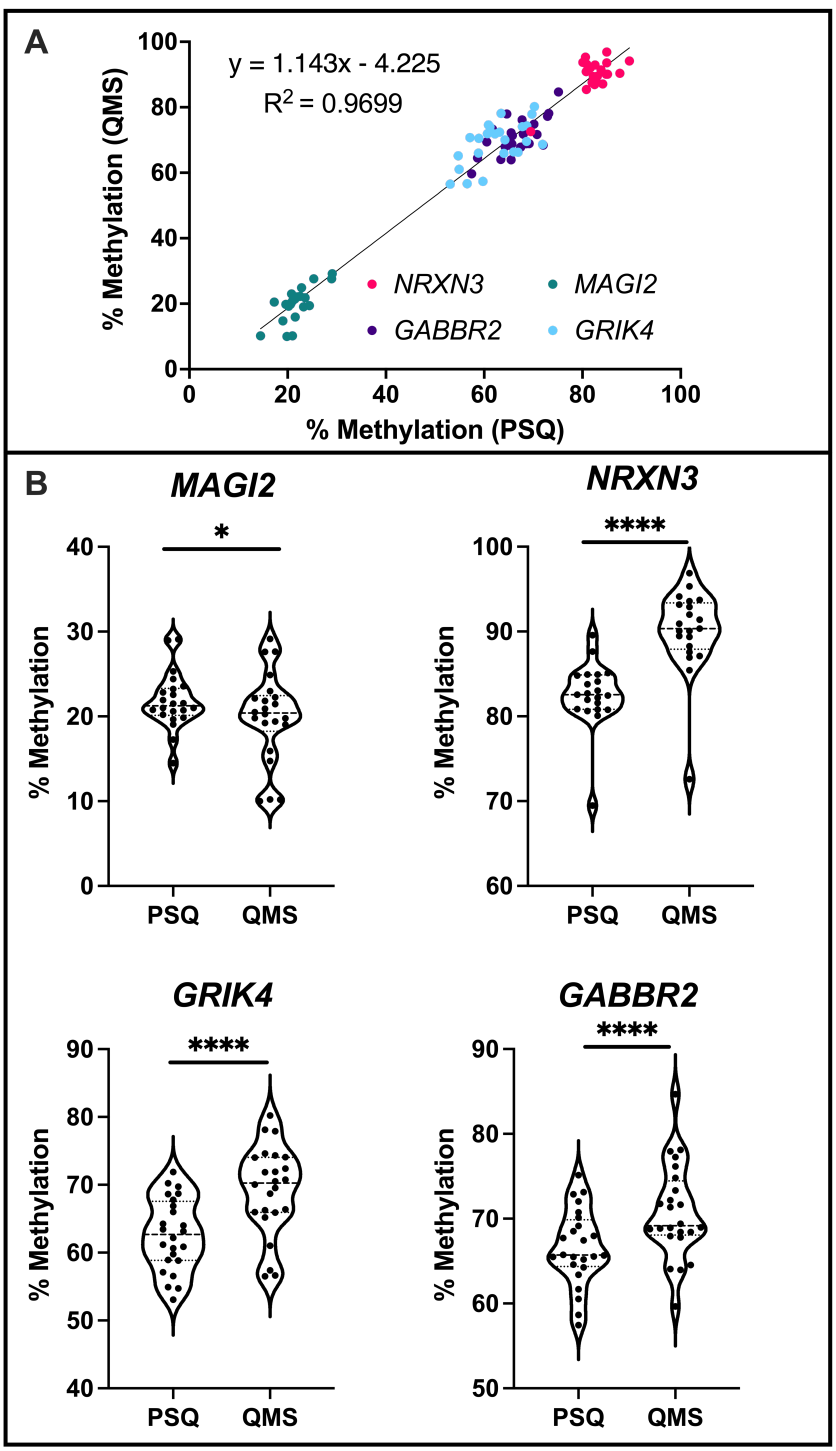
**A)** Positive correlation between methylation values using the two sequencing technologies across all four CpG sites. Statistical significance was determined by Simple Linear Regression and Pearson Correlation (p<0.0001, CC=0.98). X-axis represents percent methylation obtained via bisulfite pyrosequencing (PSQ) and the Y-axis represents percent methylation obtained via QIAseq Targeted Methyl Panel technologies (QMS). **B)** Inter-individual methylation differences for 24 individuals at CpG sites within genes *MAGI2*, *NRXN3*, *GRIK4*, and *GABBR2* as determined by PSQ and QMS. Note that y-axes are different across graphs to highlight the spread and distribution of these data at each CpG site. Statistical significance was determined with a two-tailed paired t-test (*: p<0.05, **: p<0.01, ***: p<0.001, ****: p<0.0001).

## Description

DNA methylation typically refers to the covalent bonding of a methyl group (-CH_3_) to the 5’ carbon of a cytosine residue. These methylated cytosines, referred to as 5-mC, most often precede guanines in the DNA sequence and are referred to as CpG methylation (Rakyan *et al.* 2011). DNA methylation is a critical component of the regulatory mechanisms that assure proper genome function.It plays an important role in embryonic development, X chromosome inactivation, genomic imprinting, and defense against the activity and transposition of repeat elements. Methylation is catalyzed by DNA methyltransferase enzymes (DNMTs) (Jeltsch 2006; Denis *et al.* 2011)*.* Dysregulation of DNA methylation via mutations in DMNTs, disease processes or from environmental exposures is associated with neurodevelopmental disorders, chronic disease and cancers (Greenberg and Bourc’his 2019).

Bisulfite pyrosequencing (PSQ) is one method commonly used for quantitative analysis of DNA methylation at single nucleotide level. First, DNA undergoes bisulfite modification, a chemical process that converts only unmethylated cytosines into uracils. Methylated cytosines are protected from bisulfite conversion, remain as cytosines, and thus are distinguishable from unmethylated cytosines following PCR amplification and sequencing (Bassil *et al.* 2013; Li *et al.* 2013). We previously demonstrated that this method is able to distinguish as little as 0.5% differences in DNA methylation (Nye *et al.* 2015). Its accuracy makes it an important tool for determining DNA methylation levels (Bassil *et al.* 2013; Kurdyukov and Bullock 2016). It can be used as a means to validate findings from other ‘omics technologies, including the Illumina BeadChips, methylation capture followed by sequencing, reduced representation bisulfite sequencing, and whole genome bisulfite sequencing (Roessler *et al.* 2012; Ivanov *et al.* 2013; Kulis *et al.* 2015; Murphy *et al.* 2018). Limitations of PSQ for this purpose include the need for a dedicated pyrosequencing instrument, relatively low throughput, and amplicon size limitations (generally less than 200 bp). Additionally, if there is a need to examine multiple regions of the genome, for example as might be done to assess methylation biomarker panels, designing and validating multiple assays can be costly and time consuming. Thus, new methods that offer large-scale quantitative methylation analysis at single nucleotide resolution are a pressing need.

High-throughput DNA methylation sequencing combines bisulfite conversion technology and next generation sequencing to allow for detection of 5-mC residues at single-base resolution at specific regions of interest. In the methodology used here, DNA is bisulfite converted and ligated to an adapter including a unique molecular identifier (UMI) to allow accurate methylation analysis. The regions of interest are then enriched using single primer extension (SPE) technology with a pool of primers specifically designed for the regions of interest (Xu *et al.* 2017). This technique allows for increased throughput detection of methylation simultaneously at multiple specific regions versus bisulfite pyrosequencing. The specific QIAGEN technology utilized in this study allows for the investigation of DNA methylation at multiple regions across the genome at once, many of which contain multiple CpG sites per region, allowing for detection at thousands of CpG sites concurrently. While the method utilized in this study was optimized for use on Illumina Sequencing platforms, libraries could potentially be generated for analyses on other high throughput sequencing platforms, including PacBio or Oxford Nanopore. However, the reliance on harsh bisulfite treatments that may degrade DNA in this technology may create limitations in the types of samples that can be sequenced and the necessary input amounts of DNA. The use of a bisulfite modified template with single primer extension could also introduce strand biases for specific methylation patterns during the enrichment process. Additionally, the comparability of this method to other targeted methods and its ability to detect changes in methylation have not been extensively examined.

Here we measured and compared DNA methylation at CpG sites in genes involved in neurodevelopment in umbilical cord blood from 24 individuals using bisulfite pyrosequencing and high throughput DNA methylation sequencing. Individual CpG sites in the genes *MAGI2*, *NRXN3*, *GRIK4*, and *GABBR2* were selected for this analysis. Across these four CpG sites, there was strong and statistically significant correlation between the percent methylation obtained via bisulfite pyrosequencing (PSQ) and obtained via QIAseq Targeted Methyl Panel technologies (QMS) (Figure 1A). These four CpG sites exhibit quite variable inter-individual methylation levels, showing that QMS is capable of accurately detecting levels of methylation across a wide range of potential methylation values.

At the CpG sites analyzed, there were differences between the overall levels of methylation detected by the two technologies. The range of the differences in methylation between PSQ and QMS were 0.05%-10.8% for *MAGI2*, 2.7%-14.7% for *NRXN3*, 0.1%-14.6% for *GRIK4* and 0.2%-13.3% for *GABBR2*. Across all four CpG sites, the average difference between the two technologies in detected methylation for each sample was 5.6%. The levels of methylation significantly differed between methods with QMS reporting higher overall levels of methylation at the CpG sites within *GABBR2*, *GRIK4*,and *NRXN3* and slightly lower overall levels of methylation at *MAGI2*. However, the overall spreads of the methylation distributions for the samples analyzed were remarkably similar (Figure 1B).

The results of this study demonstrate the potential of QMS to serve as an accurate and more rapid alternative to bisulfite pyrosequencing, particularly for analysis of multiple regions. Further testing is needed to corroborate these results and to better characterize the accuracy and limitations of detection of the QMS method*.* Rigorous testing of standards with known levels of DNA methylation is needed to determine the discriminatory potential across the range of possible methylation values for QMS, and further testing to determine correlations between QMS and other quantitative methylation techniques should be undertaken.

## Methods

Participants: From 2005 to 2011, prenatal clinics in Durham, North Carolina recruited Newborn Epigenetics STudy (NEST) participants to determine *in utero* exposureswhich alter the offspring’s epigenetic profiles and later health consequences*.* Pregnant, English-speaking women who were at least 18 years old were eligible to participate. This study utilized samples from 24 NEST cohort participants.

DNA Isolation: Genomic DNA was extracted from umbilical cord blood. Blood samples were collected in 10 mL ethylenediaminetetratacetic acid (EDTA) vacutainer tubes, inverted, and centrifuged to separate the leukocyte fraction (buffy coat) from the red cells and plasma. Gentra Puregene Reagents (QIAGEN) were used to isolate genomic DNA from the buffy coat, as described by the manufacturer’s protocol. Isolated genomic DNA was stored at -20ᵒC. A Nanodrop 2000 Spectrophotometer (Thermo Scientific) was utilized to determine DNA concentration and purity.

Bisulfite Pyrosequencing: Genomic DNA (800 ng) was treated with sodium bisulfite using the Zymo EZ DNA Methylation Kit (Zymo Research). PyroMark CpG Assay Design Software (QIAGEN) was used to design pyrosequencing assays. Pyrosequencing and PCR amplification were conducted using procedures described previously (Bassil *et al.* 2013). PCR amplification was optimized to produce a single robust band from amplification of 20 ng of the bisulfite modified DNA. Forward (F), reverse (R) and sequencing (S) primers were obtained from Sigma-Aldrich and their sequences are as follows: *MAGI2*, F, 5’-ATG TAG TGG GGA GGA ATA TTTTTT-3’, R, 5’-BTN-AAA CAC CAA AAC CTA AAA ACA TT-3’, S, 5’-GGG GGT TTT AGG ATT AGT-3’; *NRXN3*, F, 5’-AGT GGA ATT TTA GGG ATT TTA AAG T-3’, R, 5’-BTN-TCC AAT TAT TTC AAA ACC TCA ACT T-3’, S, 5’-GTT TTT TTT AGT GTT TAG TT-3’; *GRIK4*, F, 5’-TGG TTG TTT GTG TAG TGA GTT ATG A-3’, R, 5’-BTN-TAT TCC CAA TTA ACC TCC TTA AAC A-3’, S, 5’-TTT GTG TAG TGA GTT ATG AG-3’; and *GABBR2*, F, 5’-TTG TAG TGG GGA GTA GGG AGT ATT T-3’, R, 5’-BTN-ACA AAA AAA CCC CTC AAC CA-3’, S, 5’-TGG GGA GTA GGG AGT AT-3’. PCR was performed using the Pyromark amplification kit (QIAGEN) in a 25 µl reaction volume and thermocycler conditions as follows: 95ᵒC for 5 minutes, 50 cycles of 94ᵒC for 30s, T_m_ for 30s (*MAGI2*, 58ᵒC; *NRXN3*, 58ᵒC; *GRIK4*, 62ᵒC; *GABBR2*, 62ᵒC) and 72ᵒC for 30s with a final 10-minute extension at 72ᵒC. Defined mixtures of fully methylated and unmethylated human DNA (0%, 25%, 50%, 75%, and 100% methylation) were used in triplicate to verify that input levels of DNA methylation resulted in matching measured levels of DNA methylation with R^2^ and p-values as follows (*MAGI2*, R^2^=0.96, p= 0.004; *NRXN3*, R^2^= 0.98, p= 0.0008; *GRIK4*, R^2^= 0.97, p= 0.002; *GABBR2*, R^2^= 0.99, p= 0.0004). Following assay performance validation, samples were analyzed under the optimized conditions.

QIAseq Targeted Methylation Panel: High throughput DNA methylation sequencing was performed using the QIAseq Targeted Methyl Panel (QIAGEN) at QIAGEN’s facilities in accordance with their methodology. Briefly, 40 ng of DNA from each of the same cord blood DNA samples used for the pyrosequencing described above was bisulfite treated and simultaneously enriched for the targeted regions, according to the QIAseq Targeted Methyl Panel Library preparation. Library quality was assessed using an Agilent Bioanalyzer. Sequencing was then performed on an Illumina Miseq 2x151bp with V2 chemistry. Data were analyzed using the CLC Genomic Workbench.

Statistics: Statistical analyses were performed in GraphPad Prism. For each CpG site, two-tailed paired t-tests were performed. To assess the relationship between the two technologies across all four CpG sites, a linear regression was performed to obtain an R^2^ and p-value, as well as a Pearson Correlation.
